# Optimized oxygen therapy improves sleep deprivation-induced cardiac dysfunction through gut microbiota

**DOI:** 10.3389/fcimb.2025.1522431

**Published:** 2025-03-05

**Authors:** Shuqi Cai, Zixuan Li, Jie Bai, Yue Ding, Ruisang Liu, Liben Fang, Dengyong Hou, Sheng Zhang, Xiaohui Wang, Yujia Wang, Yuyu Jiang, Yan Xiang, Wenhui Wu, Ying He, Yunkai Zhang, Xiaomeng Ren

**Affiliations:** ^1^ College of Food Science and Technology, Shanghai Ocean University, Shanghai, China; ^2^ Naval Medical Center, Naval Medical University, Shanghai, China; ^3^ School of Life Sciences, Shanghai University, Shanghai, China; ^4^ Department of Pathogen Biology, Naval Medical University, Shanghai, China; ^5^ National Key Laboratory of Immunity & Inflammation, Naval Medical University, Shanghai, China; ^6^ Department of Cardiology, Renji Hospital, Shanghai Jiao Tong University School of Medicine, Shanghai, China

**Keywords:** sleep deprivation, cardiac dysfunction, gut microbiota, oxygen therapy, hypoxia

## Abstract

Adequate sleep is of paramount importance for relieving stress and restoring mental vigor. However, the adverse physiological and pathological responses resulting from sleep insufficiency or sleep deprivation (SD) are becoming increasingly prevalent. Currently, the impact of sleep deficiency on gut microbiota and microbiota-associated human diseases, especially cardiac diseases, remains controversial. Here, we employed the following methods: constructed an experimental sleep-deprivation model in mice; conducted 16S rRNA sequencing to investigate the changes in gut microbiota; through fecal microbiota transplantation (FMT) experiments, transplanted fecal microbiota from sleep-deprived mice to other mice; established an environment with a 30% oxygen concentration to explore the therapeutic effects of oxygen therapy on gut microbiota-associated cardiac fibrosis and dysfunction; and utilized transcriptome data to study the underlying mechanisms of oxygen therapy. The results revealed that: sleep-deprived mice exhibited weakness, depression-like behaviors, and dysfunction in multiple organs. Pathogenic cardiac hypertrophy and fibrosis occurred in sleep-deprived mice, accompanied by poor ejection fraction and fractional shortening. 16S rRNA sequencing indicated that sleep deprivation induced pathogenic effects on gut microbiota, and similar phenomena were also observed in mice that received fecal microbiota from sleep-deprived mice in the FMT experiments. The environment with a 30% oxygen concentration effectively alleviated the pathological impacts on cardiac function. Transcriptome data showed that oxygen therapy targeted several hypoxia-dependent pathways and inhibited the production of cardiac collagen. In conclusion, these results demonstrate the significance of sufficient sleep for gut microbiota and may represent a potential therapeutic strategy, where the oxygen environment exerts a protective effect on insomniacs through gut microbiota.

## 1 Introduction

In human life, every individual spends approximately one-third of our time sleeping (Sang et al., 2023). This process is indispensable for physical and psychological health, as it not only restores mental vitality but also alleviates physical fatigue (X. et al., 2024). It is widely acknowledged that sleep is intricately linked to various biological processes, and chronic sleep deprivation can have several adverse effects on human health, increasing the risk of cardiovascular disease, cancer, depression, and other illnesses (Kim et al., 2021; Liu et al., 2024; Peng et al., 2024; Zheng et al., 2024; Zhu et al., 2024). With rapid socioeconomic development and lifestyle changes, the duration and quality of sleep among modern individuals have generally declined (Bixler, 2009). The disruption of circadian rhythm balance is particularly pronounced, triggering a range of pathological processes, such as immune dysfunction and metabolic disorders, and potentially leading to premature death. Despite this, more than half of adults continue to experience sleep deprivation (Ford et al., 2015; Robbins et al., 2021), and sleep quality tends to diminish with age (McCarter et al., 2022). Even young, healthy adults can oscillate between adequate and inadequate sleep (Ferguson et al., 2021; Welsh et al., 2023). Notably, even after a period of sufficient sleep, brief episodes of sleep deprivation may still have detrimental health effects.

Oxygen is essential for sustaining the normal physiological functions of the human body. In the field of respiratory care, oxygen therapy has emerged as a fundamental and effective treatment method. It aids in improving patients’ respiratory conditions, enhances tissue oxygenation, and promotes cellular metabolism, thereby accelerating the body’s recovery process. With the rapid development of medical technology in recent years, the application of oxygen therapy has expanded significantly. As a safe therapeutic approach (N et al., 2021), oxygen therapy has demonstrated unique benefits across various medical fields. In experimental animal models, it has shown potential in inhibiting metabolic syndrome, helping to control blood glucose levels and body weight by improving insulin sensitivity and reducing fat accumulation, thus mitigating the progression of metabolic syndrome (A. and I., 2017). Furthermore, oxygen therapy has proven effective in treating hypertension, as it can alleviate the burden on the heart and reduce the risk of cardiovascular events by dilating blood vessels and lowering blood pressure (Nagatomo et al., 2010a). Most importantly, oxygen therapy has also been found to positively affect sleep quality and alleviate stress (Hadanny et al., 2024), addressing the widespread issues of sleep deprivation and excessive stress prevalent in modern society.

Increasing researches have suggested that chronic and mild inflammation is closely linked to sleep deprivation (Vgontzas et al., 1999) and the pathogenesis of stress-related diseases (F. et al., 2018). Under conditions of sleep deprivation, immune cells in the central nervous system are activated and lead to pro-inflammatory cytokine secretion, which triggers an uncontrolled inflammatory response (W. et al., 2021). These cytokines, such as tumor necrosis factor-α (TNF-α) and interleukin-6 (IL-6) (Del Gallo et al., 2014), are closely associated with sleep. Hyperbaric oxygen therapy has been shown to have a beneficial therapeutic effect on systemic inflammatory response syndrome (Oley et al., 2020). In addition, oxygen can be transported through the blood vessels via erythrocytes, and the oxygen in the plasma is called dissolved oxygen. By elevating the atmospheric pressure or increasing the oxygen concentration, the oxygen content, especially the dissolved oxygen content in the plasma, can be effectively increased (Memar et al., 2019; S, 2021), which has a positive effect on improving tissue oxygen supply, increasing tolerance to hypoxia-ischemia, and treating decompression sickness. At the same time, possible side effects of increasing oxygen concentration were also considered in this experiment. Previous studies have shown that regardless of the pressure, treatment when oxygen concentration exceeds 40% can have a detrimental effect (Nagatomo et al., 2012), whereas exposure to mild hyperbaric oxygen at the rather low oxygen concentrations (35-40% oxygen) does not lead to elevated levels of oxidative stress in rats (Nagatomo et al., 2010a, b) and humans (Ishihara, 2019).

Based on the research background outlined above, this study investigated the potential harm and underlying mechanisms of sleep deficiency on human health, especially on cardiac function. Further, we designed the adjustable oxygen chamber to control the oxygen concentration to observe the therapeutic effects of oxygen-enriched environment in SD-induced cardiac dysfunction and fibrosis through modulating the inflammatory and fibrotic response in heart tissues. To conclude, this work found the negative role of sleep deprivation in physiological health and proposed the problem-solving strategies via oxygen environment.

## 2 Materials and methods

### 2.1 Animal

6-week-old male C57BL/6J mice were purchased from Shanghai SippeBk Lab Animal Co.,Ltd (Shanghai, China). The mice were housed in a controlled environment at (23 ± 2) °C, under a standard 12h light/12h dark cycle, with unrestricted access to food and water prior to the experimental treatment. All animal experimental procedures in this study were approved by the Medical Ethics Committee of the Naval Medical Center. All operations were conducted in accordance with the Guide for the Care and Use of Laboratory Animals published by the National Institutes of Health (NIH Publication No. 85-23, revised 1985), and every effort was made to minimize animal suffering.

### 2.2 Sleep deprivation model

Sleep deprivation was conducted using a water tank-based sleep deprivation (SD) equipment, involving 18 hours of deprivation per day for 4 weeks. This equipment was purchased from Shanghai Tianhuan Tech Development Co., Ltd. (Shanghai, China). In this study, all mice, except for the blank control group, were placed on a modified sleep deprivation water platform measuring 32 cm in length and 46 cm in width, featuring 4 rows and 5 columns of compartments, each 3 cm in diameter and 5 cm in height. The water level was maintained approximately 1 cm below the platform, causing the mice to fall into the water when they fell asleep, which prompted them to wake up quickly and climb back onto the platform. The starting light time (ZT0) was consistently set at 07:00 (M. et al., 2024), regardless of the photoperiod length, and the sleep deprivation period was scheduled from ZT7 (14:00) to the following ZT1 (08:00). The animals were weighed every 4 days, and serum, organs and feces were collected at day 28.

### 2.3 Oxygen therapy

Oxygen administration treatment was conducted at the Naval Medical Center. During the experiment, after the mice completed sleep deprivation at ZT1, they were placed in an oxygen chamber (TOW-INT TECH Co, Shanghai, China), which rigorously controls the oxygen concentration in the chamber though the gas delivery equipment with high-purity oxygen and nitrogen. Different oxygen concentration (15%, 30% or 40%) was adjusted using the control panel, and the mice were treated for 2 hours. At the end of the treatment, the mice were returned to their cages for rest and subsequently subjected to sleep deprivation at ZT7 for 18 hours. The blank control group did not receive any additional interventions and was maintained under normal conditions.

### 2.4 Behavioral experiments

The equipment for the open field experiment, Morris water maze, tail suspension test, and grip strength test was provided by Shanghai Jiliang Software Tech Co. The data were analyzed using the DigBehv Animal Behavior Analysis System (Shanghai Jiliang Software Tech Co., China). The open field measured 40 cm × 40 cm, and the mice were permitted to explore the area freely for 5 minutes. The field was cleaned to eliminate any olfactory traces left by the previous mouse before the next mouse could begin the experiment. The primary observations in the open field experiment included the activity of the mice in the central area and the total distance traveled. The Morris water maze consisted of a pool with a diameter of 120 cm and a height of 45 cm, featuring a platform that measured 6 cm in diameter. A camera was fixed on a stand at a height of 210 cm above the ground, and a matching blackout curtain surrounded the setup. Before the experiment commenced, the pool was filled with water, maintaining a level approximately 1 cm below the platform. An appropriate amount of titanium dioxide was sprinkled into the water to create a uniformly white surface. On the first day of the experiment, the mice were acclimated to the water and allowed to swim while locating a visible platform within the pool, which was enclosed by the blackout curtains. This familiarization involved three consecutive training sessions, each with a 60-second cutoff. For the subsequent four days of training, the water level was adjusted to approximately 1 cm above the platform. During this period, the mice were tasked with finding the target platform using visual cues outside the pool, with four trials conducted each day (60 seconds per trial). To enhance memory retention, the starting point for each mouse’s entry into the water was randomized daily, allowing entry from each of the four quadrants of the pool. An interval of 10 minutes was maintained between trials. The system recorded the swimming speed, latency, and path of each mouse. On the sixth day, designated as the test day, the platform was removed, and the test duration was set to 120 seconds. The learning and memory capabilities of the mice were evaluated by observing the time spent in the quadrant where the platform had previously been located. The tail suspension device system can provide total observation time, activity time (struggle time), immobility time (stabilization time), and movement trajectory. The hanging tail experiment is typically conducted for 6 minutes; however, to obtain more accurate data, the first minute can be excluded from the analysis to minimize error. The levels of anxiety and depression in the mice were primarily assessed by analyzing the immobility time. The grasping force device comprised a tensiometer, a grasping net (50 mm × 50 mm), and a base. During the experiment, the mice were suspended and subsequently placed on the grasping net. The mice were then pulled backward by their tails at a constant speed, while the tensiometer recorded the grasping force exerted by the mice’s limbs in real time.

### 2.5 Detection of oxidative stress indicators

The expression levels of SOD in the hippocampus, liver, and kidney; GSH in the liver; and MDA in the kidney were measured using the following kits: T-SOD Test Kit (A001-1, Nanjing Jiancheng Biotechnology, China), GSH Test Kit (G4305, Wuhan Servicebio Technology, China), and MDA Detection Kit (G4300, Wuhan Servicebio Technology, China). The specific procedures were conducted according to the manufacturer’s instructions.

### 2.6 ELISA

Hippocampus, liver, and serum levels of TNF-α and IL-6 were measured using Mouse TNF-alpha ELISA Kit (88-7324, Thermo Fisher Scientific, USA) and Mouse IL-6 ELISA Kit (88-7064, Thermo Fisher Scientific, USA), following the manufacturer’s instructions.

### 2.7 Histopathological analysis

For histological studies, portions of the heart, liver, intestine, and lung from mice were perfused with a 4% paraformaldehyde solution at room temperature for fixation. The tissues underwent routine paraffin histology, including sectioning, H&E staining, Masson’s trichrome staining, and WGA staining. The severity of organ damage was scored on a scale of 0 to 4: 0 indicates normal tissue under study conditions, considering factors such as age, sex, and strain of the animal; 1 indicates changes just above normal; 2 indicates observable lesions that are not yet severe; 3 indicates evident lesions that are likely to be more severe; and 4 indicates very severe lesions that have compromised the entire tissue and organ (Bradley et al., 2020).

### 2.8 Cardiac enzyme profile assays

Serum was obtained, and the levels of CK, CK-MB, LDH, and LDH1 in the serum were measured using the following kits: Biobase kit #70922, Biobase kit #70923, Biobase kit #70920, and Biobase kit #70963, all from Hunan Aifang Biotechnology, China, according to the manufacturer’s instructions.

### 2.9 Echocardiography analysis

Echocardiography was conducted using an ultrasound machine (VisualSonics, Vevo 2100, Canada) equipped with a 30 MHz probe. Mice were gently anesthetized with 2% isoflurane, maintaining their heart rate between 400 and 500 beats per minute (b.p.m.). Two-dimensional cardiac images were then acquired in the parasternal long-axis view. Within this view, an M-mode cursor was positioned vertically to the interventricular septum and the posterior wall of the left ventricle at the level of the papillary muscle roots.

### 2.10 16S rRNA sequencing and analysis

The genomic DNA of the samples was extracted with the MagPure Soil DNA LQ Kit (Magan) following the manufacturer’s instructions. The V3-V4 variable region was then amplified using specific primers with barcodes and the Takara Ex Taq high-fidelity enzyme. The primer sequences used were 343F (5’-TACGGRAGGCAGCAG-3’) and 798R (5’-AGGGGTATCTAATCCT-3’) (Nossa, 2010). Subsequently, the samples were purified using AMPure XP magnetic beads. After purification, two rounds of PCR amplification and additional purification with magnetic beads were conducted. The concentration of the samples was quantified using Qubit, and the library was sequenced on the Illumina NovaSeq 6000 platform (Novogene, Beijing, China) to generate 250 bp paired-end reads. Following sequencing, the paired ends were spliced, and quality control was performed using QIIME 2 software (version 2020.10). Noise reduction and removal of chimeric sequences were carried out using the DADA2 amplicon algorithm (Callahan et al., 2016). Representative sequences of individual amplicon sequence variants (ASVs) were selected for comparative annotation against the Silva database (version 138). Bioinformatics analysis included the calculation of α-diversity indices (such as Chao1 and Shannon) for each group of samples. Principal coordinate analysis (PCoA) was performed based on the unweighted UniFrac distance matrix calculated in R to assess the β-diversity of each sample group. Additionally, analysis of variance was conducted using the ANOVA statistical algorithm in R. Differences in species abundance spectra were analyzed using the LEfSe method. Reason: Improved clarity, readability, and technical accuracy while correcting grammatical and punctuation errors.

### 2.11 Fecal microbiota transplantation assays

FMT assays were performed according to the protocols established in previous studies (Z. et al., 2021; Fang et al., 2023). Specifically, we collected small intestinal contents and thoroughly mixed them with 20% glycerol in a 1:1 ratio. The mixture was then rapidly frozen in liquid nitrogen and stored at -80°C. The samples were maintained at -80°C for a minimum of two hours. Prior to use, we diluted the samples to a working concentration of 50 mg/ml with saline and filtered them through a 70 µm cell strainer. In the experiments, each experimental mouse undergoing transplantation received 100 µl of the filtered solution daily via oral administration for two weeks, followed by a return to normal feeding for three weeks.

### 2.12 Blood oxygen detection

For oximetry measurements, a small animal pulse oximeter (Mouse Ox Plus, STARR Life Sciences) was employed. The mice were acclimated to the testing environment prior to the experiment, and they were also introduced to the neck clip to minimize stress during the procedure. This preparation aimed to prevent excessive struggling, which could lead to data loss. The test clip was carefully monitored and adjusted throughout the experiment to further reduce the likelihood of the mice becoming agitated, as this could result in data gaps or inaccuracies. The duration of the test was set to five minutes, with data recording commencing only when the system indicated that the readings were stable.

### 2.13 Western blot analysis

Protein from heart tissues was separated using a 10% SDS-PAGE gel and subsequently transferred to nitrocellulose membranes (Millipore, Germany). The membranes were probed with primary antibodies against Phospho-NF-κB p65 (Ser536) (93H1) Rabbit mAb (CST) and Phospho-p44/42 MAPK (Erk1/2) (Thr202/Tyr204) (D13.14.4E) XP^®^ Rabbit mAb (CST). Target protein levels were normalized to GAPDH (D16H11) XP^®^ Rabbit mAb (CST) as a loading control. Finally, the analysis was conducted using ImageJ software.

### 2.14 RT-qPCR analysis

Total RNA was extracted using the SteadyPure Rapid RNA Extraction Kit (Aikerui Biotechnology, AG21023, China) following the manufacturer’s protocol. The extracted RNA was subsequently analyzed using the Evo MMLV Reverse Transcription Premix Kit (Aikerui Biotechnology, AG11728, China) to reverse transcribe the RNA into complementary DNA (cDNA). Real-time quantitative PCR was conducted on a LightCycler 96 Real-Time Fluorescence PCR Detection System (Roche, Switzerland) employing the Universal SYBR qPCR Master Mix (Vazyme Biotech, Q711-02, China). The primer sequences used for RT-qPCR assays are provided in **Supplementary Table S1**.

### 2.15 RNA sequencing and analysis

Total RNA was extracted using TRIzol reagent following the manufacturer’s instructions. RNA purity and quantification were determined with a NanoDrop 2000 spectrophotometer (Thermo Scientific, USA), while RNA integrity was assessed using an Agilent 2100 Bioanalyzer (Agilent Technologies, Santa Clara, CA, USA). Transcriptome libraries were constructed using the VAHTS Universal V5 RNA-seq Library Prep kit, adhering to the provided guidelines. The libraries were sequenced using the Illumina NovaSeq 6000 sequencing platform, generating 150 bp paired-end reads. Fastp software was employed to remove low-quality reads prior to subsequent data analysis. HISAT2 software was utilized to align the reads to the reference genome, calculate gene expression levels (FPKM), and quantify gene counts using HTSeq-count. Principal Component Analysis (PCA) of gene counts was conducted in R (v 3.2.0) to assess the biological replicates of the samples. Differentially expressed genes (DEGs) were analyzed using DESeq2 software. Hierarchical clustering analysis of DEGs was performed in R (v 3.2.0) to illustrate gene expression patterns across different groups and samples. WikiPathways enrichment analyses of the differentially expressed genes were conducted based on the hypergeometric distribution algorithm. This approach was used to identify significantly enriched functional categories and to generate bar charts, chord diagrams, or enrichment analysis circle plots. Gene Set Enrichment Analysis (GSEA) was performed using GSEA software. In this analysis, a predefined set of genes was utilized, and genes were ranked according to their degree of differential expression between the two sample types. The predefined gene set was then examined to determine whether it was enriched at the top or bottom of the sorted list. The sequencing data are available in the GEO database under accession number GSE284746.

### 2.16 Data analysis

Experimental data were analyzed using GraphPad Prism software version 9.5 (USA) and are presented as mean ± standard deviation (SD). A t-test was utilized to compare two groups, while comparisons among multiple groups were performed using one-way ANOVA, followed by Tukey’s multiple comparison test for *post-hoc* analysis. Differences were deemed statistically significant when p-values were less than 0.05.

## 3 Results

### 3.1 Chronic sleep deprivation induces weakness and depression-like behaviors

**Figure 1:**
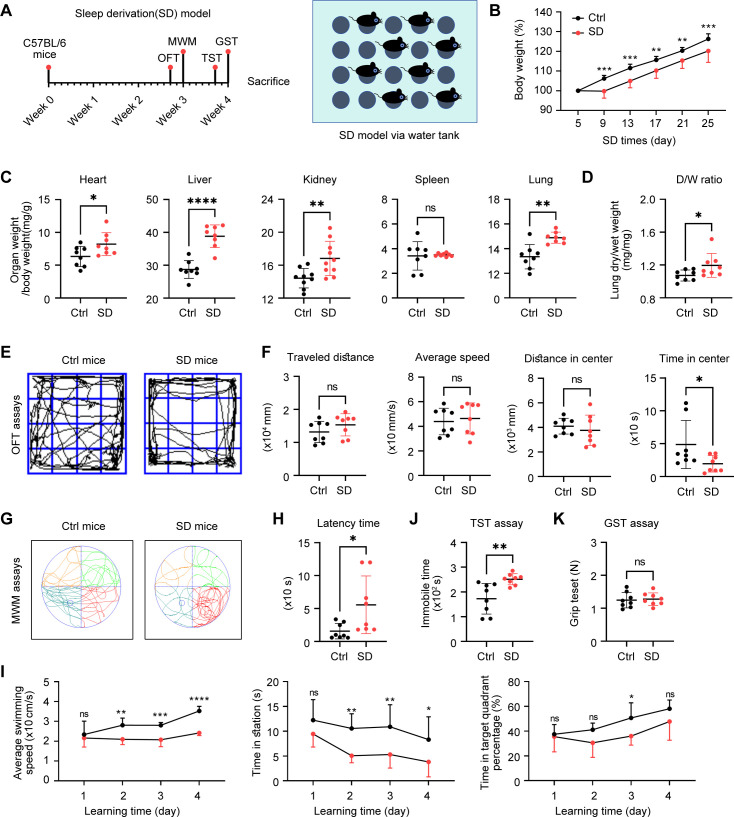
Chronic sleep deprivation (SD) induces weakness and depression-like behaviors. **(A)** Schematic diagram of sleep deprivation (SD) experiment via a water tank. **(B)** Changes of body weight in control (Ctrl) mice and SD mice during the SD experiments (n= 8 per group). **(C)** Relative organs weights in total body weight of the Ctrl mice and SD mice on day 28 as in A (n= 8 per group). **(D)** Relative lung dry weight in lung wet weight of the Ctrl mice and SD mice on day 28 as in A (n= 8 per group). **(E, F)** Representative trajectory graphs of total distance traveled during absences **(E)**, mean speed, distance traveled in the central region of activity, time spent in the central region of activity, and number of absences **(F)** in the SD model as in A (n= 8 per group). **(G-I)** Representative swimming trajectory graphs comparing the two groups in the Morris water maze **(G)**, including test day latency **(H)**, mean swimming speed, target quadrant dwell time, and percentage of time spent in the target quadrant **(I)** in the SD model as in A (n= 8 per group). **(J)** Duration of immobility in the tail suspension test (n= 8 per group). **(K)** Grip strength measured in the grip force test (n= 8 per group).Data are presented as mean ± SD; *P < 0.05; **P < 0.01; ***P < 0.001; ****P < 0.0001. Unpaired two-tailed Student’s t test **(B-D, F, H-K)**.

We generated the 4-week sleep deprivation model through the water platform-based SD equipment (**Figure 1A**). As the experiment progressed, it was observed a significant decrease in the body weight of SD mice, compared with Ctrl ones (**Figure 1B**), which might be associated with alterations in energy metabolism induced by sleep deprivation. In terms of organ weights, we calculated the weights of the heart, kidney, liver, and lung and found significant differences between the two groups, while there was no significant change in the indices of the spleen (**Figure 1C**). Further analysis showed that the lung dry wet weight ratio of the SD group mice was significantly higher than that of the Ctrl group (**Figure 1D**), which may indicate changes in lung moisture content, reflecting pathological and physiological changes in lung tissue. In behavioral tests, through the analysis of open field experiment data, we found that despite of no significant difference in motor ability between the blank group and the model group of mice, there was a significant difference in activity time in the central region (**Figures 1E, F**), indicating that sleep deprivation may affect the anxiety level of mice. In addition, in the tail suspension experiment, the immobility time of the SD group was significantly longer than that of the Ctrl group (**Figure 1J**), further confirming the anxiety behavior caused by sleep deficiency. In the Morris water maze experiment, we found significant differences in the average swimming speed and target quadrant dwell time between the two groups of mice. The Ctrl group mice were able to more accurately select the quadrant where the platform was located, with a shorter latency period and significant differences compared to the SD group (**Figures 1G-I**). These results indicated that sleep deprivation had a negative influence on the cognitive function of mice, leading to a decline in learning and memory abilities. Finally, through grip experiments, we observed that sleep deprivation did not cause changes in limb strength in mice (**Figure 1K**), indicating that this sleep interventions did not have a significant effect on muscle strength in mice. In summary, these experiments provide us with a comprehensive perspective on the physiological and behavioral effects of sleep deprivation on mice, revealing the various physiological and pathological changes that sleep deprivation may cause.

### 3.2 Chronic SD triggers systemic inflammation, oxidative stress and dysfunction in multiple organs

**Figure 2:**
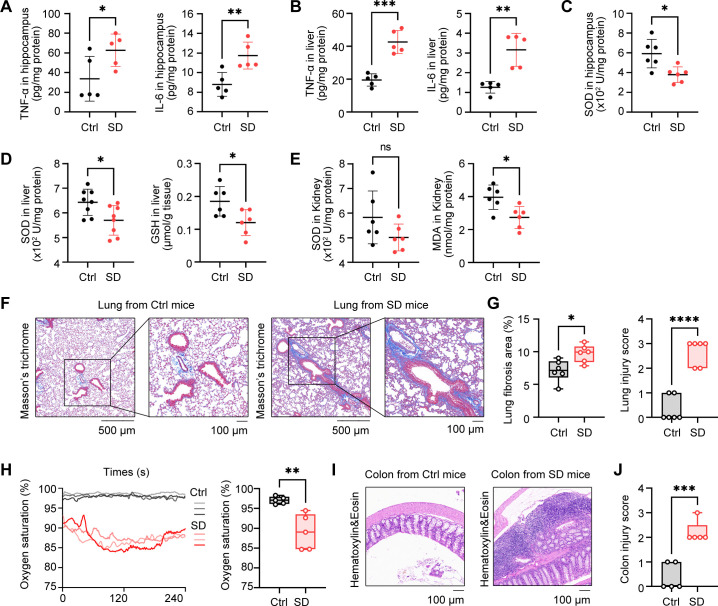
Chronic SD triggers systemic inflammation and dysfunction in multiple organs. **(A, B)** ELISA assays of TNF-α and IL-6 level in hippocampus (**A**, n=5 per group) and in liver (**B**, n=6 per group). **(C)** SOD activity in hippocampus (n=6 per group). **(D)** SOD activity and GSH concentration in liver (n=6 per group). **(E)** SOD activity and MDA concentration in kidney (n=6 per group). **(F, G)** Masson’s trichrome staining of the lungs from Ctrl mice and SD mice **(G)**. The fibrosis area and tissue pathological score was assessed (**H**, n=6 per group). **(H)** Real-time level (left) and average level (right) of oxygen saturation of Ctrl mice and SD mice, (n=5 per group). **(I, J)** H&E staining of the colons from Ctrl mice and SD mice **(I)**. The fibrosis area and tissue pathological score was assessed (**J**, n=5 per group). Data are presented as mean ± SD; *P < 0.05; **P < 0.01; ***P < 0.001; ****P < 0.0001. Unpaired two-tailed Student’s t test **(A-E, G, H, J)**.

To further characterize the adverse effects of sleep deficiency, a series of experiments were conducted to detect changes in pro-inflammatory mediators and oxidative stress indicators among various organs. Firstly, we examined the alterations in pro-inflammatory cytokines. In the hippocampus of mice subjected to SD, the levels of TNF-α and IL-6 were significantly elevated compared to Ctrl group (**Figure 2A**), indicating an inflammatory response in the hippocampus. Similarly, in the liver, the levels of TNF-α and IL-6 in the SD group were significantly higher than those in the Ctrl group (**Figure 2B**), suggesting that the livers were subjected to inflammatory damage. In terms of detecting oxidative stress indicators, we found that the expression of SOD in the hippocampus (**Figure 2C**) and liver (**Figure 2D**) of SD group mice significantly decreased, indicating a decrease in anti-oxidant activity and a weakened ability to clear free radicals, which may indicate the pathological damage. However, no significant difference was observed in the expression of SOD in the kidneys (**Figure 2E**), indicating that kidney may be less affected in this process. In addition, we found that the expression of GSH in the liver of SD group mice was significantly reduced (**Figure 2D**), which further supports the view that there may be pathological problems related to oxidative stress in the liver. It is worth noting that the expression of MDA in the kidneys was significantly reduced in the SD group, which may be related to certain types of glomerulonephritis, such as membranous nephropathy, where oxidative stress may not be the main mechanism of damage. Through Masson staining, we found more severe pulmonary fibrosis and lung injury in SD group mice (**Figures 2F, G**). The blood oxygen levels further confirmed that the lung function of SD group mice was significantly worse than that of Ctrl group (**Figure 2H**), indicating that sleep deprivation may cause significant damages to lung tissue. Finally, by observing the intestinal tissue and liver of mice through HE staining, we found that the colon tissue of SD group mice showed significant inflammatory response (**Figure 2I**) and obvious tissue injuries (**Figure 2J**), and livers were also subjected to inflammatory damages (**Supplementary Figures S1A, B**). In summary, sleep deprivation may lead to a series of pathological changes by triggering inflammatory responses and oxidative stress in multiple organs, thereby affecting the health status of mice.

### 3.3 Pathological cardiac fibrosis and dysfunction in SD mice

**Figure 3:**
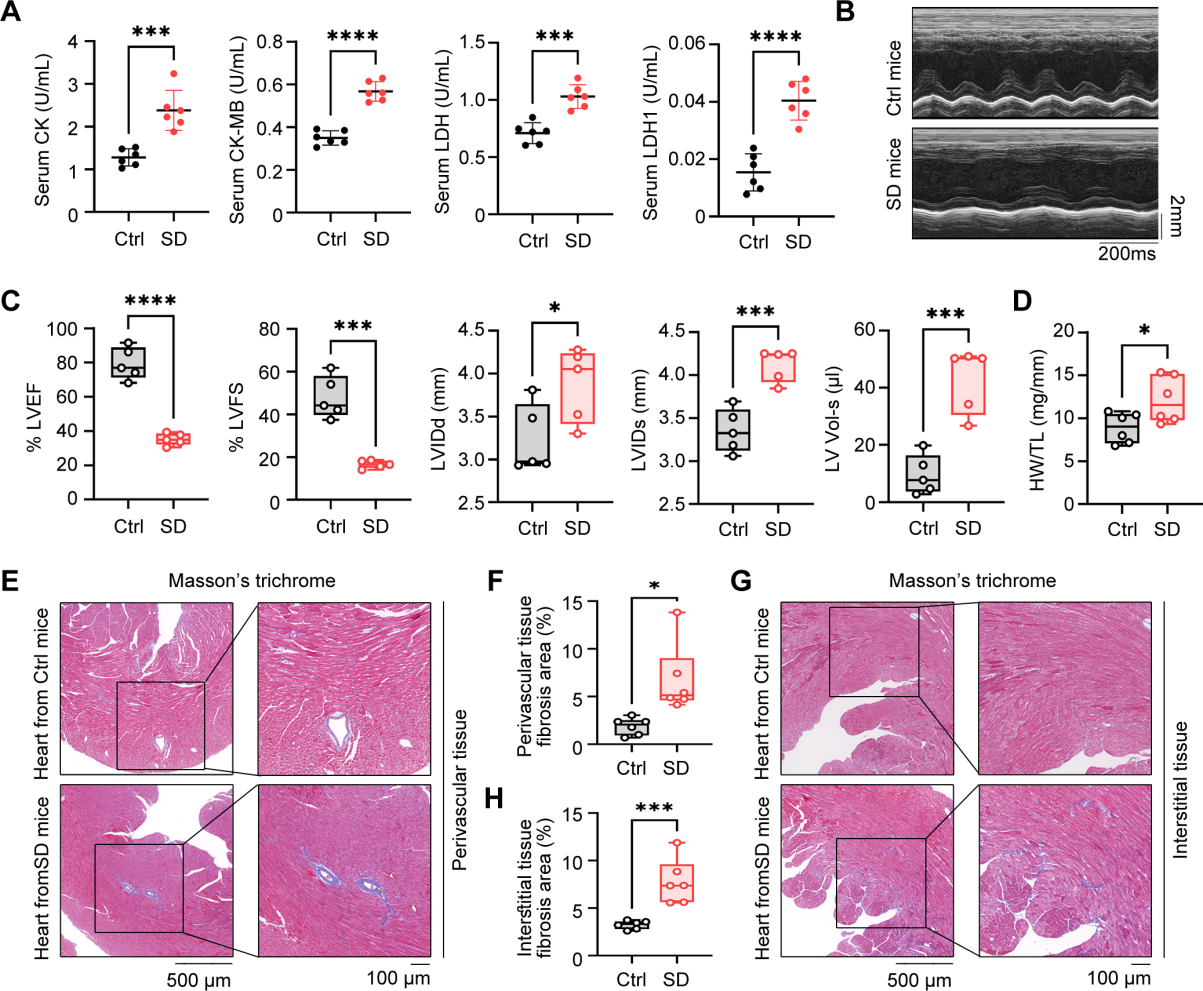
Pathological cardiac fibrosis and dysfunction in SD mice. **(A)** Serum level of CK, CK-MB, LDH and LDH1 in Ctrl mice and SD mice (n=6 per group). **(B)** Representative echocardiographic M-mode images of left ventricles from Ctrl mice and SD mice. **(C)** Echocardiographic measurement of the LVEF, LVFS, LVIDd, LVIDs and LV Vol-s of Ctrl mice and SD mice (n =5 per group). **(D)** The ratio of heart weight to tibia length (HW/TL) of Ctrl mice and SD mice (n =5 per group). **(E, F)** Representative Masson’s trichrome images **(E)** and quantitation of perivascular tissue **(F)** fibrosis in myocardial tissues from Ctrl mice and SD mice (n =6 per group). **(G, H)** Representative Masson’s trichrome images **(G)** and quantitation of interstitial tissue **(H)** fibrosis in myocardial tissues from Ctrl mice and SD mice (n =6 per group). Data are presented as mean ± SD; *P < 0.05; **P < 0.01; ***P < 0.001; ****P < 0.0001. Unpaired two-tailed Student’s t test **(A, C, D, F, H)**.

To investigate the impact of SD on cardiac function, we firstly assessed the cardiac injury by measuring serum myocardial enzyme spectrum indicators. These indicators, including CK, CK-MB, LDH, and LDH1, are well-acknowledged sensitive markers of cardiac injury. We observed that, compared to the Ctrl group, the serum levels of myocardial enzyme spectrum indicators in the SD group mice were significantly elevated (**Figure 3A**), which suggested that myocardial cells may have sustained damage, resulting in compromised cell membrane integrity and the subsequent release of myocardial enzymes into the bloodstream. The impact of SD on cardiac function was further evaluated using echocardiography technology. By analyzing the indicators of systolic and diastolic function of the heart, it was observed that the heart function of SD group mice was significantly worse than that of Ctrl group (**Figures 3B–D**). The deterioration of these indicators further confirms the potential damaging effect of SD on the structure and function of the heart. To confirm cardiac injury from a histological perspective, we performed Masson staining on the cardiac tissue which can clearly display the deposition of collagen fibers in cardiac tissue, thereby evaluating the degree of fibrosis. By comparing the cardiac tissue sections of the SD group and the Ctrl group, we found that SD group exhibited significant fibrosis in both the perivascular area (**Figures 3E, F**) and the interstitial tissues (**Figures 3G, H**). This result further supports the view that SD causes cardiac damage, indicating that chronic sleep deprivation may trigger changes in cardiac structure, thereby affecting cardiac function. Summing up the above, we found that chronic SD has a significant damaging effect on cardiac structure and function.

### 3.4 Sleep deprivation induces pathogenic alterations in gut microbiota

**Figure 4:**
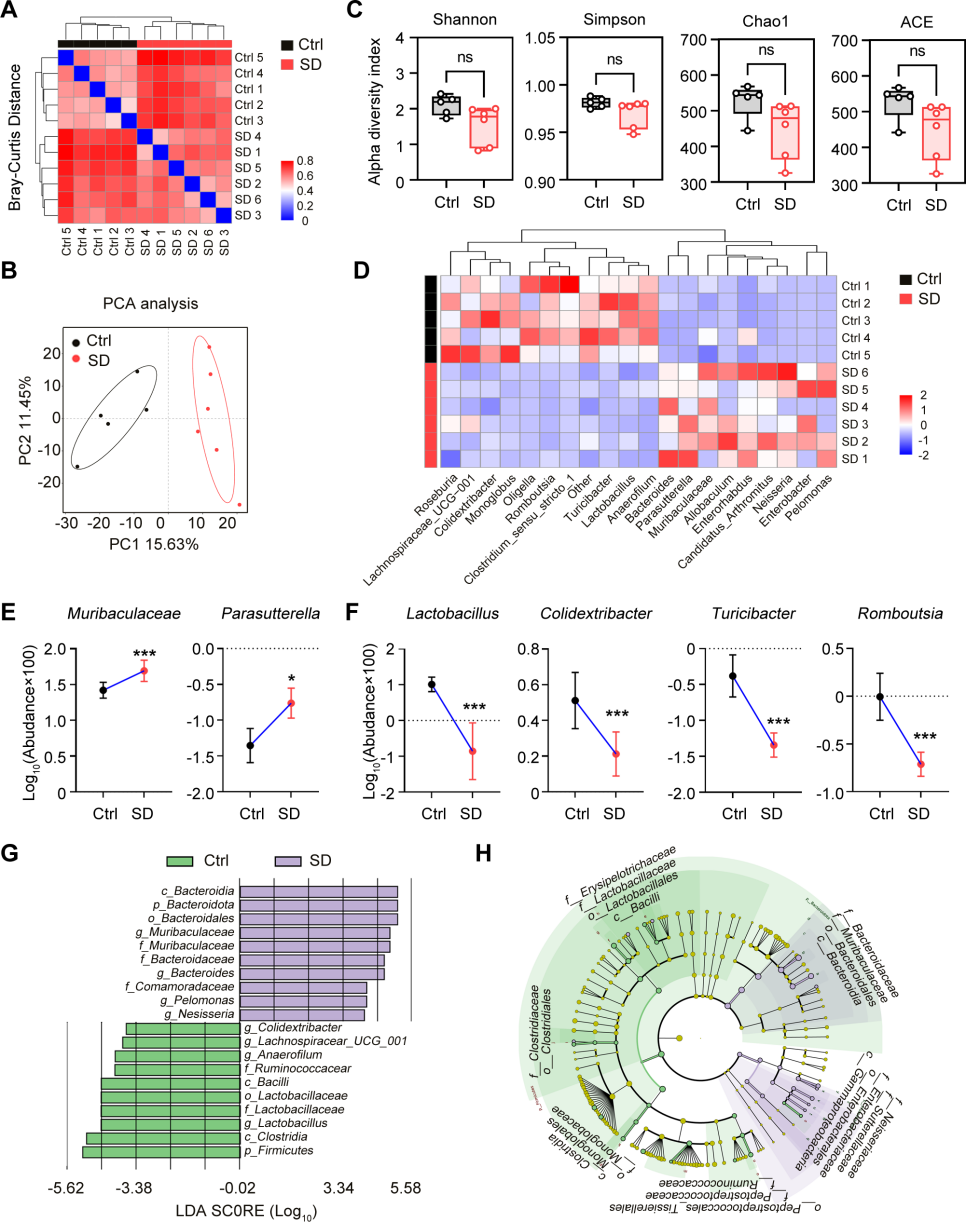
Gut microbiota changes caused by SD. **(A)** Heatmap showing the Bray-Curtis Distance between Ctrl mice and SD mice. **(B)** PCA of the data showed in **(A)**. **(C)** α-Diversity indexes, including Shannon, Simpson, Chao1, and ACE of the data shown in **(A)**. **(D)** Heatmap of the differential genera of gut microbiota in Ctrl mice and SD mice. **(E, F)** Relative abundance of the indicated gut microbiota in Ctrl mice and SD mice. **(G)** LDA algorithm of Ctrl vs SD gut microbiota bacterial genera with an LDA score threshold of 2.0. **(H)** Cladogram of the main taxa of micro-biota in Ctrl mice and SD mice based on LEfSe analysis. Data are presented as mean ± SD; *P < 0.05; **P < 0.01; ***P < 0.001; ****P < 0.0001. Unpaired two-tailed Student’s t test **(C, E, F)**.

In this study, we previously observed a significant inflammatory response in the colon tissues. To further investigate the role of SD on the composition and function of gut microbiota, we collected gut contents from both Ctrl group and SD group and performed high-throughput sequencing analysis of 16S rRNA. By conducting ANOSIM on the sequencing results, we found the significant difference in gut microbiota between Ctrl group and SD group (**Figure 4A**). This indicates that SD has a substantial impact on the structure of gut microbiota. In addition, the PCA analysis further confirmed the significant changes in gut microbiota caused by SD (**Figure 4B**), showing the separation trend of microbial composition between the two groups. To evaluate the diversity of microbial communities, we conducted alpha diversity analysis, including Shannon, Simpson, Chao 1, and ACE index. These analysis results revealed fewer differences in microbial species abundance and diversity between Ctrl group and SD group (**Figure 4C**). In terms of changes in specific bacterial groups, we found that *Muribaculaceae* and *Parasottella* bacteria significantly increased after SD, which is closely linked to damaged intestinal barrier function, which facilitated the entry of pathogens and harmful factors from the intestine into the circulation system (Mo et al., 2022; Yang et al., 2023; Tian et al., 2024). At the same time, beneficial bacteria such as *Lactobacillus*, *Colidextribacter*, *Turicibacter*, and *Romboutsia* were significantly reduced after SD (**Figures 4D–F**). The reduction of beneficial bacteria is widely acknowledged to result in the overwhelming proliferation of gut pathogenic bacteria, impaired immune defense function, and even systemic chronic inflammatory response. The reduction in these bacteria is often associated with an increased risk of digestive system diseases and cardiovascular system (Rastogi and Singh, 2022; Wu et al., 2022; Zhang et al., 2023). We next used LEfSe method to determine the specific microbial community associated with SD. In the SD group, the Bacteroid genus was the most common harmful bacteria in the feces, while in the Ctrl group, several probiotics dominated, including *Colidextribacter* and *Lactobacillaceae* (**Figures 4G, H**), further emphasizing the significant differences in gut microbiota between the two groups. In summary, these results indicate substantial differences in gut microbiota between the SD and Ctrl groups, suggesting that gut microbiota and intestinal inflammation might be the pathogenic factors in weakness and organs dysfunction induced by sleep deficiency.

### 3.5 Transplantation fecal microbiota from SD mice results in cardiac fibrosis and dysfunction

**Figure 5:**
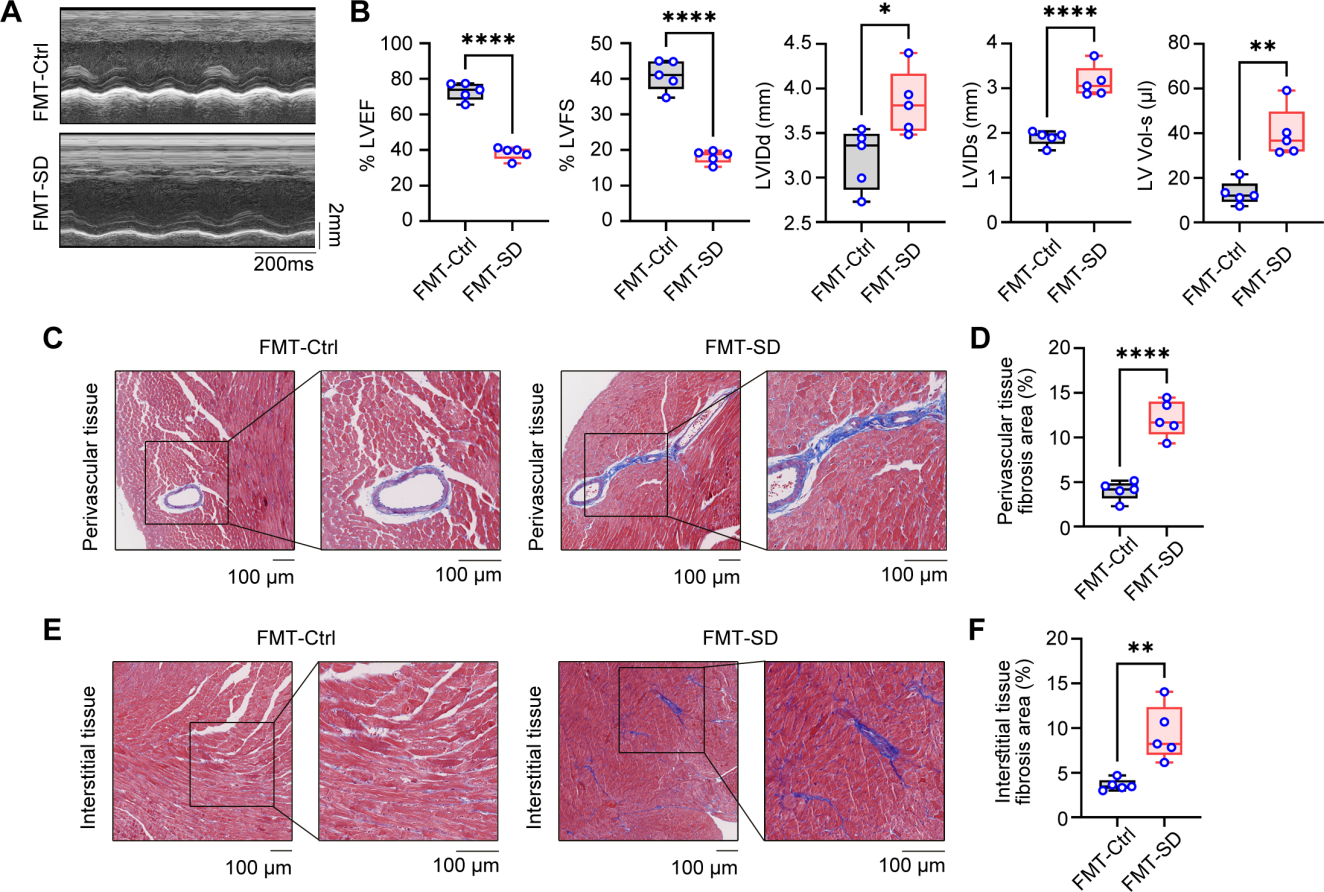
Fecal microbiota transplantation from SD mice results in cardiac fibrosis and dysfunction. **(A)** Representative echocardiographic M-mode images of left ventricles from the mice colonized by fecal microbiota of Ctrl mice and SD mice. **(B)** Echocardiographic measurement of the LVEF, LVFS, LVIDd, LVIDs and LV Vol-s of mice as in A (n =5 per group). **(C, D)** Representative Masson’s trichrome images **(C)** and quantitation of perivascular tissue **(D)** fibrosis in myocardial tissues from mice as in A (n =5 per group). **(E, F)** Representative Masson’s trichrome images **(E)** and quantitation of interstitial tissue **(F)** fibrosis in myocardial tissues from mice as in A (n =5 per group). Data are presented as mean ± SD; *P < 0.05; **P < 0.01; ***P < 0.001; ****P < 0.0001. Unpaired two-tailed Student’s t test **(B, D, F)**.

The above findings indicate that the gut microbiota of mice subjected to SD model was out of balance and included several pathogenic bacteria species. To further investigate the effects of these gut microbiota on cardiac function, fecal microbiota transplantation (FMT) assays were employed using the intestine contents of SD mice as the transplant source. After three weeks of transplantation, echocardiographic analysis revealed that the heart function of mice receiving intestine contents transplantation from SD mice (FMT-SD) was impaired, while no obvious pathological results were observed in mice receiving feces from Ctrl mice (**Figures 5A, B**). Additionally, using the Masson staining technique to analyze cardiac fibrosis, we observed that FMT-SD mice exhibited more pronounced fibrosis in the perivascular area (**Figures 5C, D**) and interstitial tissues (Figures 5E, F). Next we planned to investigate the effects of normal fecal on improving the cardiac function of SD mice (**Supplementary Figure S2A**). Our histopathological analysis by Masson’s trichrome staining and serological test of CK, LDH, IL-6 and TNF-α concentration all demonstrated that feeding SD mice with normal fecal material had no therapeutic effects (**Supplementary Figures S2B–E**). These findings suggest that the harmful gut microbiota of SD mice, such as *Muribaculaceae* and *Parasutterella*, may contribute to pathological cardiac fibrosis and impaired cardiac function in normal mice. These data also demonstrated that sleep deprivation had an adverse effect on cardiovascular system in a gut microbiota-dependent manner.

### 3.6 The therapeutic effects of oxygen chamber under different oxygen concentration on SD mice

**Figure 6:**
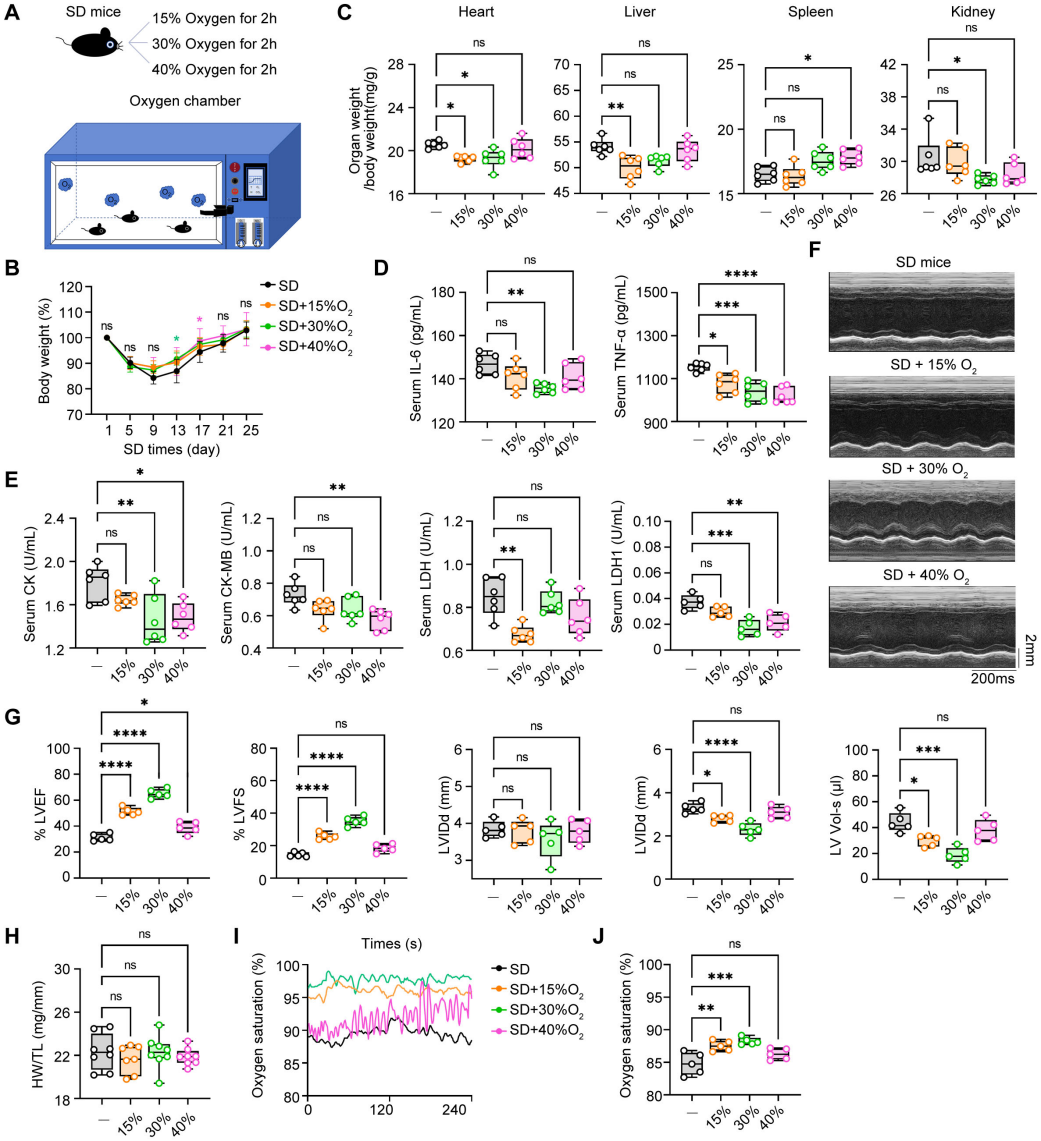
The therapeutic effects of oxygen chamber under different oxygen concentration in SD mice. **(A)** Schematic diagram of the therapeutic strategy via oxygen chamber. **(B)** Changes of body weight in SD mice treated with different oxygen concentration during the SD experiments (n= 8 per group). **(C)** Relative organs weights in total body weight of the mice as in B (n= 8 per group). **(D)** ELISA assays of TNF-α and IL-6 level in serum from the mice as in B (n=6 per group). **(E)** Serum level of CK, CK-MB, LDH and LDH1 from the mice as in B (n=6 per group). **(F)** Representative echocardiographic M-mode images of left ventricles. **(G)** Echocardiographic measurement of the LVEF, LVFS, LVIDd, LVIDs and LV Vol-s of mice as in B (n =5 per group). **(H)** The ratio of heart weight to tibia length (HW/TL) of mice as in B (n =8 per group). **(I, J)** Real-time level **(I)** and average level **(J)** of oxygen saturation of mice as in B (n =5 per group). Data are presented as mean ± SD; *P < 0.05; **P < 0.01; ***P < 0.001; ****P < 0.0001. One-way ANOVA **(B-E, G-J)**.

Oxygen therapy has been widely accepted to relieve the sickness and fatigue in many medical treatments, however, its role in sleep deprivation and SD-associated cardiovascular diseases are largely obscure. In the way, we generated the oxygen chamber for adjustable oxygen concentration with real-time monitoring (**Figure 6A**). Next, we administered SD mice in this oxygen chamber for 2 hours every day with different concentrations of oxygen (15%, 30%, and 40%) and observed the therapeutic effects. First, we found that all three concentrations of oxygen therapy reversed the weight loss of SD mice to some extent (**Figure 6B**). In addition, compared with the SD mice without oxygen treatment, the oxygen treatment groups with concentrations of 15% and 30% had an effect on reducing the proportion of the indicated organs in total body weight, including the heart, liver, and kidney, indicating that these two concentrations of oxygen therapy may have a potential protective effect on these organs function. However, the oxygen group with a concentration of 40% showed no significant effect on the relative organ weight, which may indicate that high-concentration oxygen therapy did not have a significant improving effect on these organs (**Figure 6C**). Then we analyzed the circulating pro-inflammatory cytokines in the serum to investigate the role of oxygen therapy in SD-triggered systemic inflammation. ELISA assays demonstrated that administering oxygen at an concentration of 30% significantly reduced TNF-α and IL-6 levels after SD, compared to the other two groups (**Figure 6D**). This suggests that oxygen therapy at a concentration of 30% may have a inhibitory effect on pro-inflammatory cytokines production, helping to alleviate the inflammatory response during sleep deprivation. Serum analysis of myocardial enzyme spectrum indicators also found that administering oxygen at a concentration of 40% significantly increased LDH1 compared to the SD group, indicating that high-concentration oxygen therapy may aggravate the damage of myocardial cells. Otherwise, oxygen therapy at concentrations of 15% and 30% can reduce the expression of myocardial damage-associated enzymes to a certain extent (**Figure 6E**). Finally, we further observed the effect of oxygen therapy via echocardiography, indicating that the treatment effect was more significant in the oxygen group with a concentration of 30% (**Figures 6F–I**). In addition, we also measured blood oxygen concentration and found that the oxygen saturation rate of mice from 30% oxygen-treated group was higher than that of the SD group as well as other oxygen concentration groups (**Figures 6J, K**). To sum up, it is safe to arrive at the conclusion that oxygen therapy through modulating the environmental oxygen concentration exerted an effective protective influence on sleep deficiency-induced inflammatory response and pathological injuries, and identified 30% oxygen concentration had the most best therapeutic effects among these different concentrations.

### 3.7 Oxygen therapy effectively improved the cardiac inflammation and fibrosis in SD mice.

**Figure 7:**
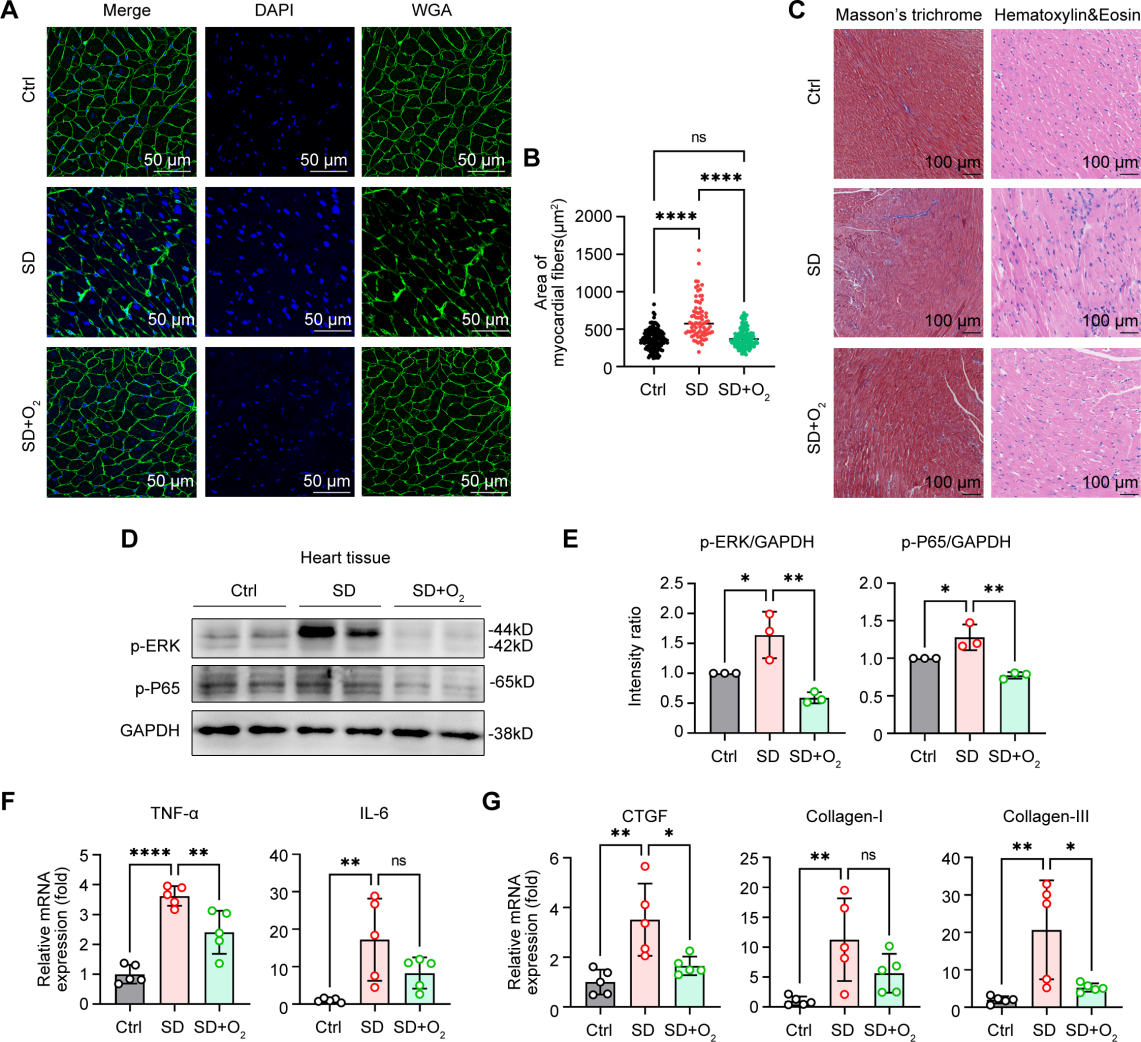
Oxygen therapy effectively improved the cardiac inflammation and fibrosis in SD mice. **(A, B)** WGA images of the hearts **(A)** and the area of myocardial fibrosis **(B)** from Ctrl mice, SD mice and SD mice treated with 30% oxygen treatment. **(C)** Masson’s trichrome staining (left) and H&E staining (right) of the hearts from the mice as in **(A)**. **(D, E)** Immunoblot assays of phosphorylated (p)-ERK, p-P65 and GAPDH **(D)** and the quantitation **(E)** of these protein in the hearts from the mice as in **(A)**. **(F, G)** Q-PCR assays of the indicated pro-inflammatory cytokines **(F)** and fibrotic genes **(G)** in the hearts from the mice as in A (n=5 per group). Data are presented as mean ± SD; *P < 0.05; **P < 0.01; ***P < 0.001; ****P < 0.0001. One-way ANOVA **(B, E-G)**.

First and foremost, we assessed the safety of 30% concentration of oxygen treatment in normal mice *in vivo*. We found that oxygen environmental at 30% had little adverse effects on the cardiac structure, function and tissue damage (**Supplementary Figures S3A–C**). In addition, Q-PCR assays of cardiac tissues from normal mice administrated with 30% oxygen treatment found that this therapy also did not affect the expression of pro-inflammatory genes and collagen genes in the mice heart (**Supplementary Figures S4A, B**). However, the 40% oxygen treatment resulted in the increase of pathogenic genes expression in cardiac tissues, indicating this concentration might not be the candidate strategy for ameliorating the tissue damage of hearts from SD mice. We next further analyzed the protective effects and underlying mechanisms of 30% concentration of oxygen treatment on cardiac dysfunction. Compared with mice from the Ctrl group, we observed that the mice from the SD group exhibited pronounced symptoms of myocardial hypertrophy, characterized by a significant increase in the area of myocardial fibers, suggesting sleep deprivation resulted in severe pathological changes in the cardiac structure. Notably, when these mice were administrated with 30% oxygen concentration, the symptoms of myocardial hypertrophy were alleviated to some extent, and the myocardial fiber area was reduced accordingly (**Figures 7A, B**). In previous studies, we have demonstrated that SD treatment leads to a significant increase in the lesioned area of cardiac fibrosis. Intriguingly, it is observed that treatment with 30% oxygen concentration was effective in alleviating the undesirable cardiac fibrosis during sleep deprivation (**Figure 7C**). By analyzing the results of HE staining, we observed that the treatment also reduced the inflammatory cell infiltration to some extent (**Figure 7C**). Furthermore, immunoblot assays of proteins from heart tissues showed that sleep deprivation triggered the activation of nuclear factor kappa-B (NF-κB) and mitogen-activated protein kinase (MAPK) signal pathways, indicated by the increased phosphorylation of P65 and ERK, which suggested that SD treatment not only led to changes in the structure of the heart, but also influenced immune function, making mice more susceptible to infection by external viruses. As expected, we found oxygen therapy effectively reversed SD-induced phosphorylation and P65 and ERK, the markers for the activation of NF-κB and MAPK signal pathways (**Figures 7D, E**). In addition, we also found that the mRNA levels of pro-inflammatory cytokines TNF-α and IL-6 in the heart tissues of mice were significantly increased after SD treatment compared with the Ctrl group, while treatment with 30% oxygen concentration significantly reduced the levels of these pathogenic inflammatory mediators (**Figure 7F**). Similarly, the mRNA levels of several fibrosis-related markers CTGF, Collagen-I, and Collagen-III also increased during SD model, but decreased in SD mice treated with oxygen therapy (**Figure 7G**). In this part, we concluded the treatment with 30% oxygen concentration can effectively alleviate the symptoms of myocardial hypertrophy, inflammation and fibrosis in the SD mice, which provides a strong experimental basis for further cardioprotective strategies.

### 3.8 Oxygen therapy reversed the harmful effects of sleep deprivation on cardiac hypoxia metabolism and collagen response

**Figure 8:**
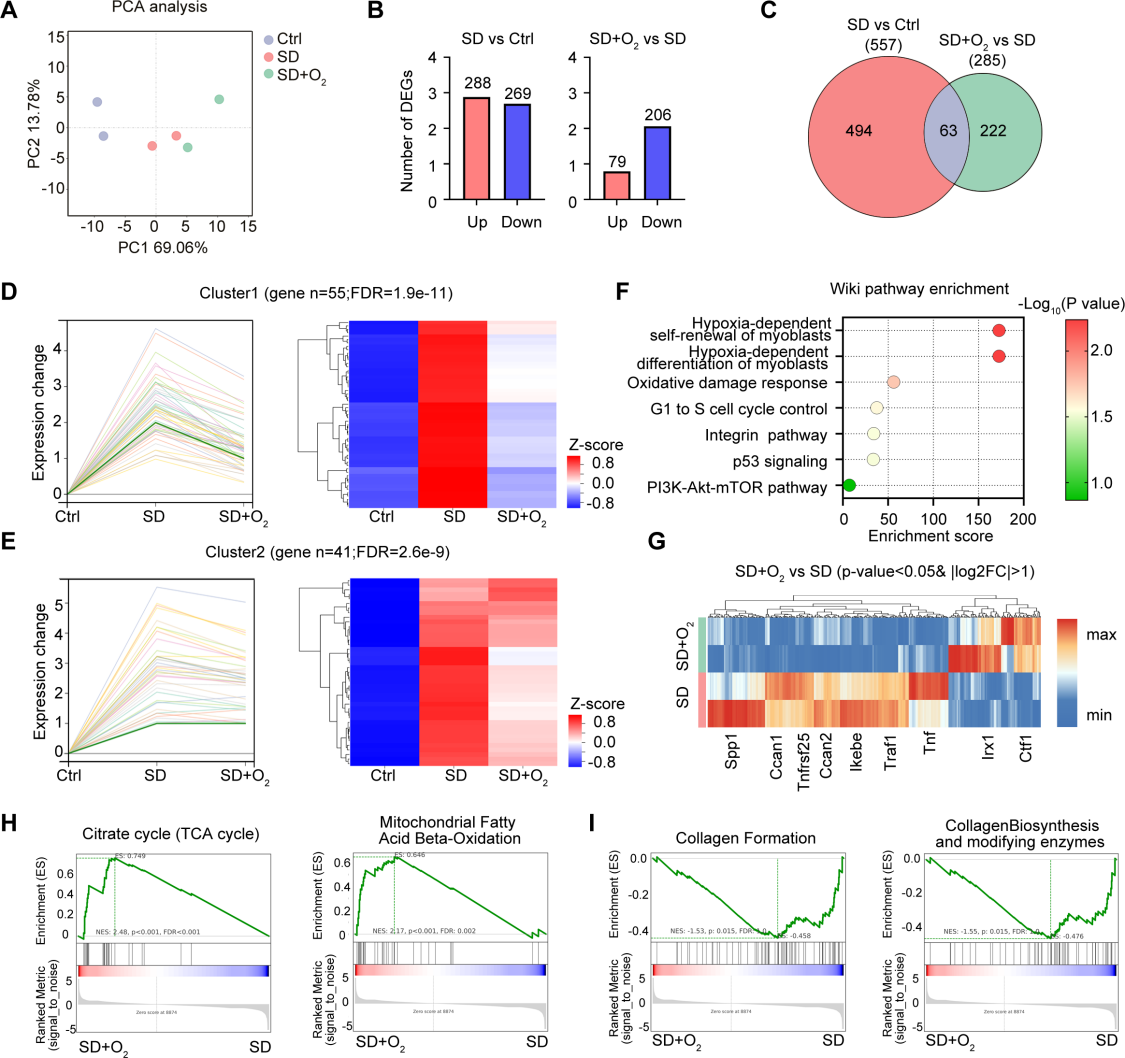
Oxygen therapy reversed the harmful effects of sleep deprivation on cardiac hypoxia metabolism and collagen response. **(A)** PCA analysis of the transcriptome data from the hearts of Ctrl mice, SD mice and SD mice treated with 30% oxygen (n=2 per group). **(B)** The number of DEGs between SD mice vs Ctrl mice (left), and between SD mice treated with 30% oxygen vs SD mice (right) in the transcriptome data. **(C)** Veen diagram showing the overlay DEGs as in **(B)**. **(D, E)** Cluster analysis of gene differentially expressed in Ctrl mice, SD mice and SD mice treated with 30% oxygen, including cluster 1 **(D)** and cluster 2 **(E)**. **(F)** Wiki pathways of the gene enriched in cluster 1 and cluster 2. **(G)** Heatmap of the DEGs between the hearts from SD+O_2_ and SD mice. **(H, I)** GSEA analysis the up-regulated pathways **(H)** and down-regulated pathways **(I)** between the hearts from SD+O_2_ and SD mice.

To further investigate the potential effects of oxygen therapy on cardiac function in mice, we conducted a systematic transcriptome sequencing analysis on three groups: the Ctrl group, the SD group, and the 30% oxygen therapy group (SD+O_2_). The PCA scatter plot revealed significant alterations in the gene expression profile at a global level among the heart tissues from Ctrl, SD and SD+O_2_ mice (**Figure 8A**). Specifically, a total of 288 genes were upregulated, while 269 genes were downregulated following SD treatment. This change indicates that SD treatment may trigger the great alternations in expression of genes associated with cardiovascular disease, leading to various pathological states (Jancsó et al., 2018). Notably, oxygen therapy resulted in 79 gene expression up-regulation and 206 gene expression down-regulation (**Figure 8B**), suggesting that oxygen therapy may help reverse gene expression alternations in the heart after SD treatment. Of the 63 genes shared between the three groups (**Figure 8C**). We divided these differentially-expressed genes into different clusters and further focused on the genes whose expression were induced in SD group and were further down-regulated by oxygen therapy, mainly including Cluster 1 and Cluster2 (**Figures 8D, E**). Through Wiki pathway enrichment analysis, we found that several hypoxia-dependent pathways was the most significantly relevant pathway in oxygen therapy treatment (**Figure 8F**). This result reflects the importance of oxygen in the regulation of cardiac function. In addition, other important pathways were also influenced by oxygen therapy, involving the cell cycle regulation, oxidative damage and integrin pathway, indicating the extensive role of oxygen in cell cycle, inflammation and oxidative metabolism (**Figure 8F**). We next analyzed the specific functional gene expression through heatmap analysis. It is observed that SD treatment up-regulated the expression of genes involving inflammatory mediators, chemokines, and oxidative stress, including *Arg1*, *Spp1*, *Ccl8*, *Ccl22*, *Hmox1* (**Supplementary Figure S5**). Meanwhile, the expression of genes encoding epigenetic enzymes, *Dnmt3b* and *Kdm6a* was downregulated. Interestingly, SD treatment resulted in the inducible expression of *Nr1d1*, the key transcription factor in circadian clock, and the inhibitory expression of Bmal1, indicating the endogenous network between sleep deficiency and circadian clock-associated genes (**Supplementary Figure S2**). Next, we focused the influence of oxygen therapy in the hearts from SD mice. Oxygen therapy effectively inhibited the expression of genes involving in inflammation and immune cell function, including *Spp1*, *Ccan1*, *Tnfrsf25*, *Ccan2*, *Lkebe*, *Traf1*, and *Tnf*, while the expression of *Lrx1* and *Ctf1* was up-regulated (**Figure 8G**). Changes in these genes may be closely related to the protective effect of oxygen therapy on the cardiac dysfunction and fibrosis. What’s more, Gene Set Enrichment Analysis (GSEA) analysis was performed to characterize the global influence of oxygen therapy in SD hearts. It turned out that oxygen therapy promoted the several oxygen-associated cellular metabolism pathways, including tricarboxylic acid (TCA) cycle and mitochondrial fatty acid β-oxidation (**Figure 8H**). Otherwise, oxygen therapy inhibited the expression involving in formation of collagen and collagen biosynthesis and modifying enzymes (**Figure 8I**), which also verified our previous findings that oxygen treatment effectively improved the cardiac fibrosis during SD. In this part, we utilized the transcriptome data to give a detailed description of the protective effect of oxygen therapy in cardiac metabolism, inflammation and fibrosis, and further confirmed the significance of sufficient oxygen supply in cardiac function during sleep deprivation (**Supplementary Figure S6**).

## 4 Discussion

The main findings of this paper are as follows: (1) Sleep deprivation (SD) negatively impacts the learning and memory abilities of mice, affects cognitive function, induces anxiety, and adversely affects cardiac function, leading to tissue lesions; (2) Compared to the SD group, oxygen therapy at concentrations of 15% and 30% can improve cardiac function in mice, with the effects being more pronounced at the 30% concentration; (3) Oxygen therapy at a concentration of 30% effectively mitigates inflammation and fibrosis by SD. Based on these results, we conclude that SD resulted in inflammation and fibrosis, caused fibrotic lesions in heart tissue, and that oxygen therapy at a concentration of 30% is effective in reducing these inflammatory and fibrotic changes in heart tissue.

Due to the rapid development of society and the increasing pressures of modern life, sudden deaths resulting from sleep deficiency and deprivation have become more frequent worldwide in recent years. Insufficient sleep had been reported to disrupt the heart’s circadian rhythm, elevating the risk of sudden death (Stangherlin et al., 2021). Additionally, sleep deprivation-associated anxiety can diminish work efficiency, distract attention, and increase the likelihood of safety accidents, potentially leading to depression (Dupuis et al., 2023). While there have been significant advances in the treatment of sleep deprivation, most have been treated with medications. While medications can help improve sleep deprivation or relieve these sufferings, oxygen therapy is more likely to have long-term benefits. First of all, oxygen therapy is a non-invasive treatment that does not place any additional burden on the body and does not produce drug side effects (X et al., 2021). Second, oxygen therapy can provide rapid relief of symptoms, allowing people to return to their normal lives and work more quickly. In addition, oxygen therapy can also help people establish a healthier lifestyle, which can prevent the recurrence of sleep deprivation and anxiety symptoms. Of course, despite the many advantages of oxygen therapy, medication can provide a deeper understanding and treatment of the root cause of symptoms, especially in some serious diseases where medication is necessary. However, at the same time, the disadvantages of drug treatment are also obvious, such as drug side effects, dependence, and drug withdrawal reactions. In contrast, oxygen therapy is a safe, stable, and reversible treatment modality that is more suitable for long-term use. At present, hyperbaric oxygen therapy has achieved remarkable results in many fields (Chang et al., 2020), but the study of oxygen therapy under atmospheric pressure is also of far-reaching significance. First, atmospheric oxygen therapy has a wide range of applications. Many diseases, such as trauma, burns, infections, etc., do not necessarily require oxygen therapy in a high-pressure environment. In these cases, normobaric oxygen therapy is not only convenient and easy, but can also be performed at the patient’s home, greatly improving the convenience and feasibility of treatment. Secondly, atmospheric oxygen therapy also plays an important role in improving the quality of life of the general healthy population, which can promote the utilization of oxygen in the human body and improve the body’s metabolic efficiency, thereby helping to improve the overall health level of the general healthy population. Furthermore, studying oxygen therapy under atmospheric pressure can help us better understand the mechanism of oxygen therapy. Although hyperbaric oxygen therapy has shown powerful effects in some cases, its mechanism of action remains largely unknown. By studying oxygen therapy at atmospheric pressure, we can gain a deeper understanding of the physiological effects of oxygen, which can provide more evidence for future research and clinical applications.

The objective of this study was to investigate the therapeutic effects of varying concentrations of oxygen therapy on sleep-deprived (SD) mice under normal pressure conditions, as well as the pathophysiological characteristics and potential mechanisms associated with oxygen therapy in the context of sleep deprivation. To simulate a real-life sleep-deprived population, we developed an animal model of sleep deprivation lasting 28 consecutive days. In this model, mice were subjected to 18 hours of sleep deprivation using a modified water platform. After the SD model was constructed, the mice were tested through a series of behavioral experiments. The results are consistent with previous studies in which we found that sleep deprivation significantly reduced the learning and memory ability of mice, caused cognitive biases, and increased anxious behaviors (Liu and Chen, 2019; W et al., 2024). In order to explore the organ pathologies that sleep deprivation may cause, we conducted a detailed pathophysiological study of the heart, lungs, liver, intestines, and kidneys of mice. The results of biochemical analysis showed that compared with the Ctrl group, the serum concentration of CK, CK-MB, LDH and LDH1 in the SD group were significantly increased. In addition, the expressions of pro-inflammatory cytokines TNF-α and IL-6 in the liver increased significantly, while anti-antioxidant molecules GSH and SOD decreased. The similar phenomenon was also observed in the hippocampus. The expression of MDA in the kidneys was significantly decreased. More importantly, we found the pathological changes in the intestinal tissues in SD mice. We next observe the harmful effects of SD on cardiac structure and function, including cardiac ejection function. And the fibrosis area was increase in cardiac tissues from SD mice. Using 16S rRNA sequencing, we found sleep deprivation led to the great change in gut microbiota composition and proportion, with pathogenic bacteria increased and beneficial bacteria decreased. The abundance of pathogenic bacteria in gut microbiota from SD mice such as *Muribaculaceae* and *Parasutterella* is closely linked to an imbalance in intestinal immune homeostasis and damaged intestinal barrier function, which facilitated the entry of pathogens and harmful factors from the intestine into the circulation system (Mo et al., 2022; Yang et al., 2023; Tian et al., 2024). On the other hand, a significant decrease in beneficial bacteria, including *Lactobacillus* and *Romboutsia*, is widely acknowledged to result in the overwhelming proliferation of gut pathogenic bacteria, impaired immune defense function, and even systemic chronic inflammatory response. The reduction in these bacteria is often associated with an increased risk of digestive system diseases and cardiovascular system (Rastogi and Singh, 2022; Wu et al., 2022; Zhang et al., 2023). In this way, the severe intestinal structure damage made it possible for these pathogenic gut microbiota do harm to whole body organs through circulating system. Fecal microbial transplantation (FMT) experiments showed that feces from SD mice were able to cause heart dysfunction and fibrosis in normal mice. However, we fed SD mice with normal fecal material had no therapeutic effects. We inferred that it was possible that the pathogenic bacteria, including *Muribaculaceae* and *Parasutterella*, in SD mice fecal material did cause the adverse cardiac injury through impairing gut barrier and facilitating circulating immune factors, however, supplementation of beneficial bacteria such as *Lactobacillus* had little effects. Previous studies (Jiang et al., 2022) have shown that long-term changes in lifestyle and other life factors can lead to changes in the gut microbiota of human beings, but there is still a relative lack of research on whether sleep deprivation causes heart inflammation or fibrosis. As the core of the blood circulatory system, the normal function of the heart directly affects the blood and oxygen supply to other organs in the body. Cardiovascular disease is one of the most common diseases in modern society, including coronary heart disease, hypertension, arrhythmia, etc., which are closely related to changes in heart function and structure. Therefore, an in-depth study of the pathophysiological changes of cardiac tissue is of great significance for understanding the pathogenesis of related diseases, as well as for prevention, diagnosis and treatment. In this study, SD mice were treated with oxygen at concentrations of 15%, 30%, and 40%, and the protective effects were observed. We observed that a 30% concentration of oxygen therapy increased the body weight of mice and improved in serum levels of CK, CK-MB, LDH, LDH1. The blood oxygen saturation and the serum inflammatory cytokine levels of IL-6 and TNF-α also verified the therapeutic effects of 30% oxygen therapy in sleep sufficiency-mediated dysfunction and diseases. In addition, we also found the potential harmful effects of 40% oxygen treatment with elevated expression of pro-inflammatory cytokines and collagen genes in cardiac tissues of normal mice, indicating too high a dose of oxygen treatment might not be candidate therapeutic strategy to treat SD-associated cardiac dysfunction.

We further investigated the effects and underlying molecular mechanisms of oxygen therapy at a concentration of 30% on cardiac function in SD mice. Specifically, cardiomyocytes exhibited pathological hypertrophy under SD conditions, and the relative mRNA expression of TNF-α, IL-6, CTGF, Collagen-I and Collagen-III in cardiac tissues were significantly elevated. Concurrently, the phosphorylation of P65 and ERK were also increased, indicating the activation of inflammatory and fibrotic processes. Following oxygen therapy at a concentration of 30%, the condition of cardiomyocytes improved significantly; the aggregation of inflammatory cells diminished, and the expression levels of the aforementioned inflammation- and fibrosis-related mediators in cardiac tissue were markedly reduced. Additionally, the activation of NF-κB and MAPK signal pathways decreased, suggesting that oxygen therapy has anti-inflammatory and anti-fibrotic effects. In order to further understand the effects of oxygen therapy at the global transcriptome level, we performed RNA-seq of cardiac tissues in the Ctrl group, SD group, and SD+O_2_ group. By comparing the differential genes between the three groups, we found that a subset of the differential genes that were significantly up-regulated after oxygen therapy were associated with metabolic disorders. Using the Wiki pathway analysis, we found that the hypoxia-dependent pathways were the most significantly influenced pathway after oxygen therapy, which was closely related to cardiac metabolism and fibrosis processes. We also found several gene expression alternations including important circadian clock core genes *Bmal1* and *Nr1d1*, and epigenetic modifying enzymes *Kdm6a* and *Dnmt3b*. Future studies need to further clarify the cross-talk between sleep deprivation and circadian clock-associated epigenetic regulation of cardiac functions, which might contribute to a deeper understanding of the pathogenesis of sleep deficiency-mediated diseases and is expected to provide more precise diagnosis and treatment options.

In summary, this study found the improving effects of oxygen therapy under atmospheric pressure conditions on cardiac function in SD mice and elucidated its underlying molecular mechanisms. Our results demonstrated that 30% concentration oxygen therapy effectively improves cardiac inflammation and fibrosis, indicating that the heart is one of the organs susceptible to inflammatory damage following sleep deprivation, which was attributed to gut microbiota changes and that oxygen therapy holds significant therapeutic potential. These findings provide a theoretical foundation for future clinical applications and may offer new directions for intervention strategies in sleep-related cardiovascular diseases.

## Data Availability

The RNA sequencing data have been deposited to the Gene Expression Omnibus (GEO) database (https://www.ncbi.nlm.nih.gov/geo/) via its standard submission process with the accession number GSE284746.
