# Supplementary material for: Optimized oxygen therapy improves sleep deprivation-induced cardiac dysfunction through gut microbiota
Source: Front Cell Infect Microbiol. 2025 Mar 5;15:1522431. doi: 10.3389/fcimb.2025.1522431 (PMC11919660; doi:10.3389/fcimb.2025.1522431)
Supplement: Supplementary file 1 [file DataSheet1.pdf]

Supplementary Material

1 Supplementary Figures and Tables

1.1 Supplementary Figures

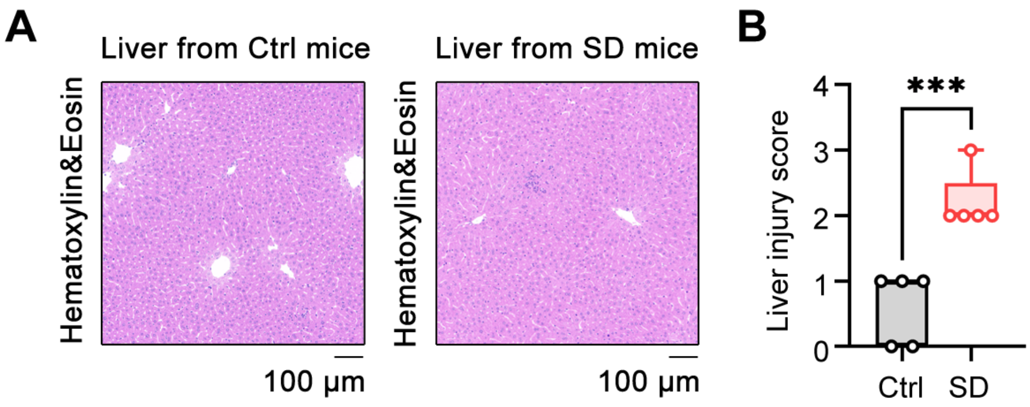

Supplementary Figure 1. Chronic SD triggers inflammatory injury in livers.

(A-B) H&E staining of the livers from Ctrl mice and SD mice (A). The tissue pathological score was assessed (B, n=5 per group).

Data are presented as mean ± SD; *P < 0.05; **P < 0.01; ***P < 0.001. Unpaired two-tailed Student's *t* test (B).

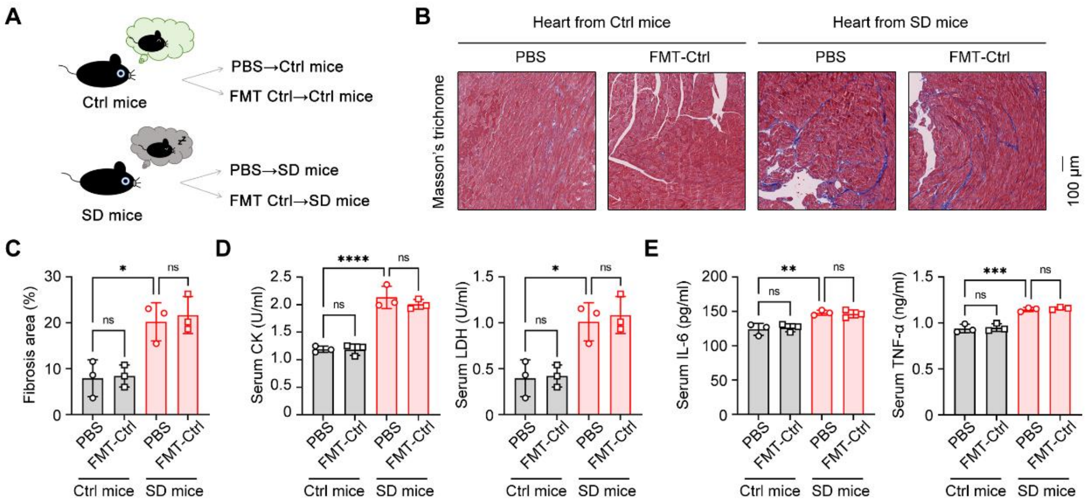

Supplementary Figure 2. The effects of normal fecal microbiota transplantation on the cardiac function of SD mice.

(A) Schematic diagram of Ctrl and SD mice administrated with normal fecal microbiota transplantation or PBS control.

(B-C) Representative Masson's trichrome images (B) and quantitation of tissue fibrosis (C) in myocardial tissues from Ctrl mice and SD mice treated as in **A** (n =3 per group).

(D-E) Serum level of CK and LDH (D), IL-6 and TNF-α (E) in Ctrl mice and SD mice treated as in **A** (n=3 per group).

Data are presented as mean ± SD; *P < 0.05; **P < 0.01; ***P < 0.001****P < 0.0001. One-way ANOVA (C-E).

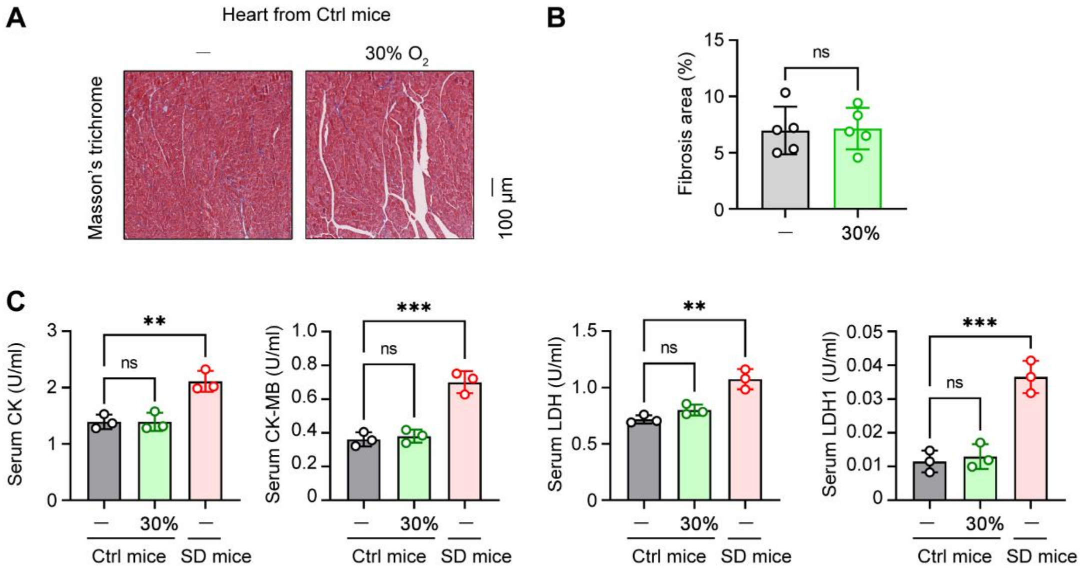

Supplementary Figure 3. The safety of 30% oxygen therapy in mice.

(A-B) Representative Masson's trichrome images (A) and quantitation of fibrosis tissue (B) in myocardial tissues from Ctrl mice administrated with 30% oxygen therapy or not (n =5 per group).

(C) Serum level of CK, CK-MB, LDH and LDH1 in Ctrl mice administrated with 30% oxygen therapy or not, and in SD mice as the positive control (n=3 per group).

Data are presented as mean ± SD; *P < 0.05; **P < 0.01; ***P < 0.001. Unpaired two-tailed Student's t test (B) and One-way ANOVA (C).

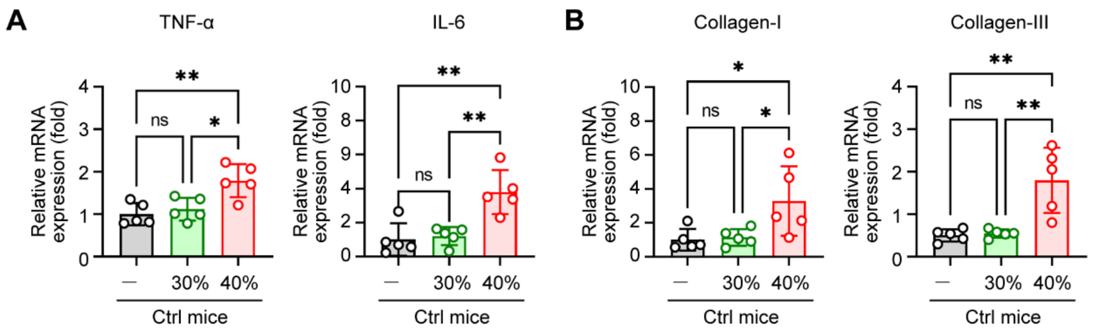

Supplementary Figure 4. The influence of 30% and 40% oxygen therapy in normal mice.

(A-B) Q-PCR assays of the indicated pro-inflammatory cytokines (A) and collagen genes (B) in the hearts from the Ctrl mice administrated with 30%, 40% oxygen therapy or not (n=5 per group).

Data are presented as mean ± SD; *P < 0.05; **P < 0.01. One-way ANOVA (A and B).

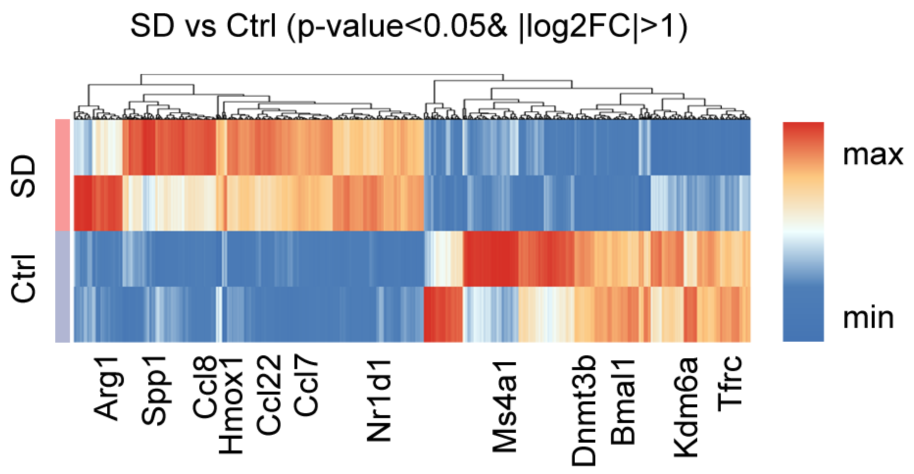

Supplementary Figure 5. The transcriptome data of the hearts from Ctrl mice and SD mice.

Heatmap of the DEGs between the hearts from Ctrl mice and SD mice.

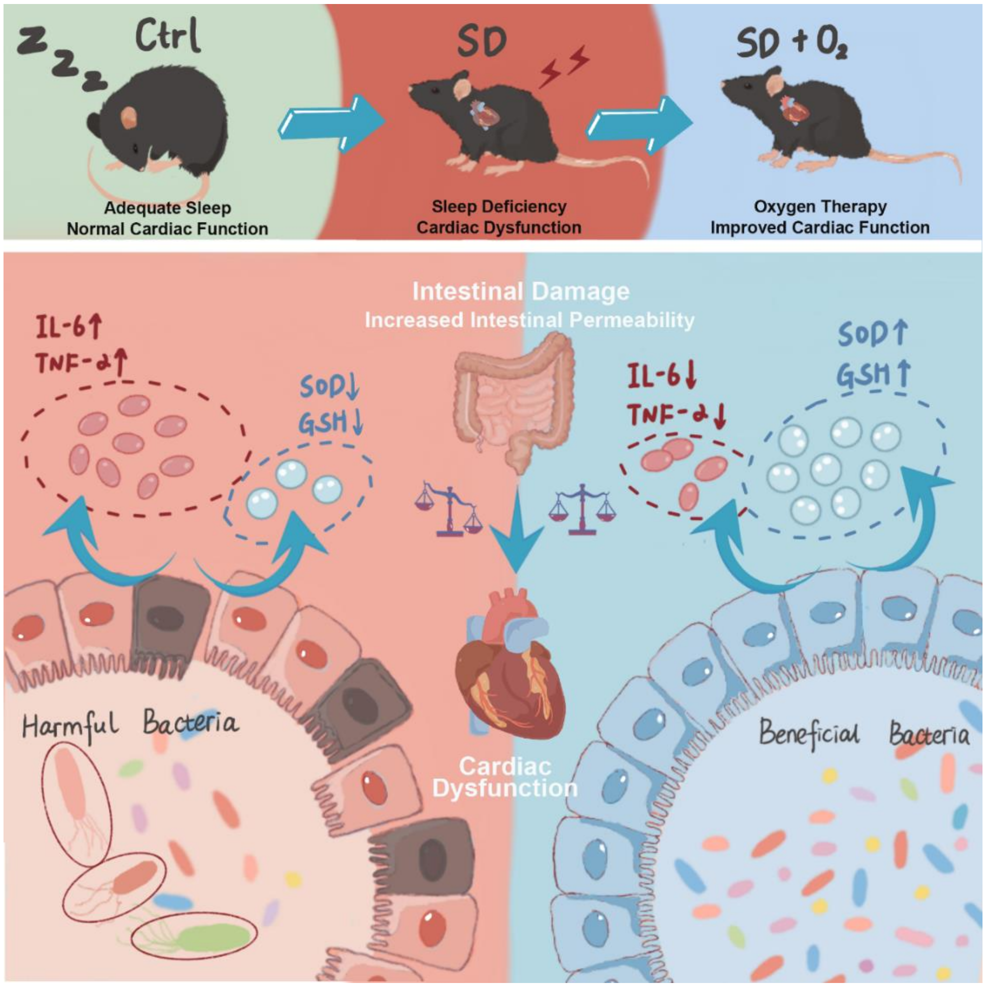

Supplementary Figure 6. Working model of the optimized oxygen therapy on the adverse effects of cardiac dysfunction by sleep deprivation.

This study demonstrated that the pathogenic effects of sleep deprivation on murine cardiac function in an experimental model, which was closely related to imbalance of gut microbiota and overwhelming abundance of harmful bacteria such as Muribaculaceae and Parasutterella. These alternation of gut microbiota resulted in systemic chronic inflammatory response, characterized by the increase of pro-inflammatory cytokine production and the decrease of anti-oxidant molecules. Through an optimized oxygen therapy at 30% concentration for 2h daily, the improved cardiac function and obvious reduction of cardiac fibrosis and collagen formation were observed in SD mice. Our study provided an novel therapeutic strategy of delivering sufficient oxygen to individuals suffering from sleep deficiency or sleep deprivation, which exhibited effective improvement of their cardiovascular systems.

1.2 Supplementary Tables

Table 1. Primers used for RT-qPCR

Gene name	Forward primer	Reverse primer
Tnf-mouse	GCATGGATCTCAAAGACAACCA	ATGGGCTCATACCAGGGTTT
Il6-mouse	AGACAAAGCCAGAGTCCTTCA	CTTGGTCCTTAGCCACTCCT
Ctgf-mouse	GGGCCTCTTCTGCGATTTC	ATCCAGGCAAGTGCATTGGTA
Col1α1-mouse	TGCTAACGTGGTTCGTGACCGT	ACATCTTGAGGTCGCGGCATGT
Col3α1-mouse	ACGTAAGCACTGGTGGACAG	CCGGCTGGAAAGAAGTCTGA
